# MaizeSNPDB: A comprehensive database for efficient retrieve and analysis of SNPs among 1210 maize lines

**DOI:** 10.1016/j.csbj.2019.10.003

**Published:** 2019-11-07

**Authors:** Wei Zhou, Lei Wang, Wenming Zheng, Wen Yao

**Affiliations:** a National Key Laboratory of Wheat and Maize Crop Science, College of Life Sciences, Henan Agricultural University, Zhengzhou 450002, China; b College of Life Sciences, Xinyang Normal University, Xinyang 464000, China; c National Key Laboratory of Crop Genetic Improvement and National Centre of Plant Gene Research (Wuhan), Huazhong Agricultural University, Wuhan 430070, China

**Keywords:** Maize, SNP, Database, Genomic variation, Genotype, Graphical interface

## Abstract

With the rapid decreasing of sequencing cost, large volume of genotype data has been generated in many organisms based on high-throughput sequencing, which was utilized in various fields of biological studies in the post-genome era. The raw sequencing data were usually deposited in the NCBI SRA database. Construction of the database to store and analyze the processed genotype data is an essential step for the utilization of the genotype data by the community. Up to now, a comprehensive genotype database is still missing from maize, which is an important crop of the world. We report the construction of the MaizeSNPDB database using genotype data of 1210 maize line across 35,370,939 SNP sites refined from a large set of genomic variations reported by the maize HapMap 3 project. We further implemented several genetic analysis programs as graphical interfaces in the MaizeSNPDB database. SNPs in user-specified genomic regions could be easily extracted and analyzed in MaizeSNPDB. The whole dataset and code of MaizeSNPDB is available at https://github.com/venyao/MaizeSNPDB. MaizeSNPDB is deployed at http://150.109.59.144:3838/MaizeSNPDB/ for online use. The MaizeSNPDB database is of great value to future maize functional genomic studies, which can also facilitate marker-assisted breeding in maize.

## Graphical abstract

**Figure:**
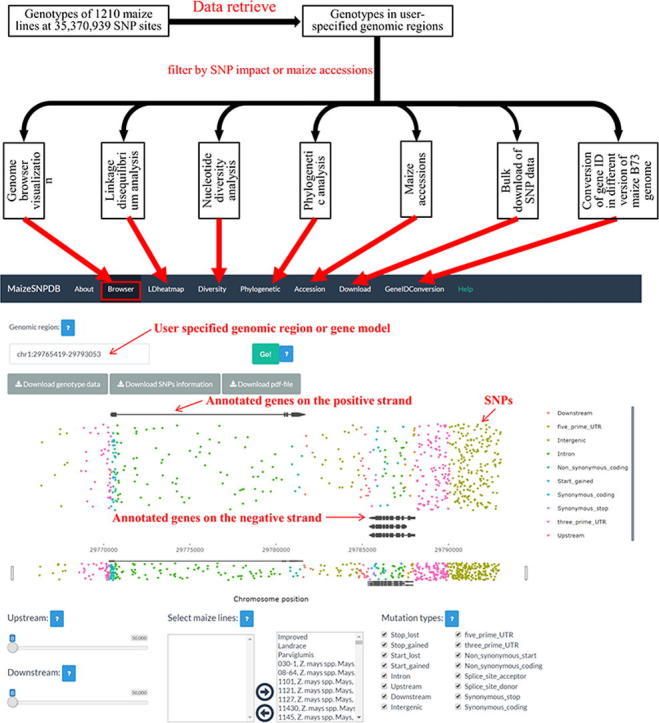

## 1 Introduction

Genomic variation is an important force in evolution. Identification of genomic variations between different individuals is the key to the understanding of phenotype variations and genetic regulations [1]. SNP is the predominant type of genomic variation and is utilized in various type of biological studies, including genome-wide association studies (GWAS), quantitative trait loci (QTL) mapping, genetic prediction, population genomics studies, marker-assisted breeding, etc. SNP data of more and more organisms were reported as the sequencing cost is getting lower with the rapid development of next-generation sequencing technology [2], [3], [4], [5]. Efficient storage of SNPs in database with analysis tools has been proved to be helpful to the functional genomics studies of many organisms. Numerous databases had been constructed to store and analyze SNP data of various organisms [6], [7], [8].

Maize (*Zea mays* subsp. *mays*) is one of the most important crops in the world. In maize, tens of millions of SNPs were reported in many genome-wide association studies, which is a powerful approach to dissect the genetic basis of various agronomic traits [9], [10], [11]. More than 83 million variant sites in maize were reported by the maize HapMap 3 project based on the whole-genome sequencing data of 1218 maize lines publicly available [2]. The generated dataset was deposited in public repository for downloading, which is the most comprehensive genomic variation dataset for maize up to now. A stringent computational pipeline was developed by an international consortium of maize research groups to construct this dataset based on publicly-available genome sequencing data of a large set of maize lines. However, utilization of the whole dataset or genotype data in specific genomic regions are challenging for biologists without professional coding skills. A web-based tool named SNPversity was recently developed for retrieve of SNP data in specified genomic regions based on this dataset [7]. This database is of value to the functional genomic studies of maize, although only a simple query interface is provided. The maize reference genome used in SNPversity is B73 version RefGen_V3, which is outdated as the latest maize reference genome widely used is version RefGen_V4 updated using single-molecule real-time sequencing [12]. The gene models were fully updated in RefGen_V4.

In this study, we constructed a comprehensive database containing 35,370,939 SNP sites among 1210 maize lines based on the data reported by the maize HapMap 3 project [2]. A genome browser and several other tools are provided for efficient extraction and analysis of SNPs in user-specified genomic regions.

## 2 Results

### 2.1 Data acquisition and storage

**Supplementary Figure 1:**
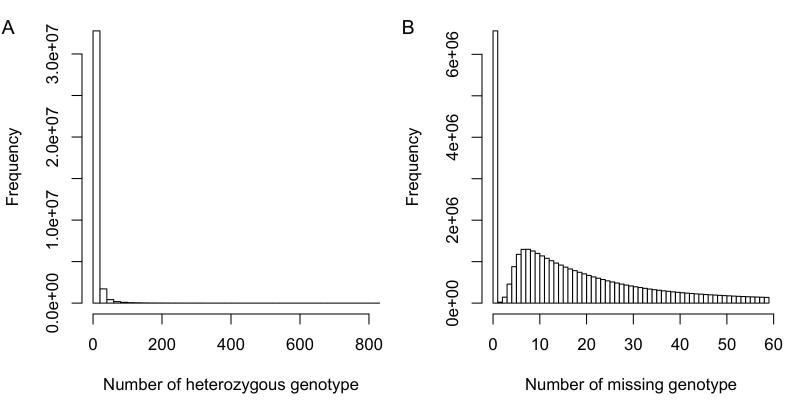
Frequency of the number of heterozygous genotype and missing genotype at 35,370,939 SNP sites among the 1210 maize lines.

**Supplementary Figure 2:**
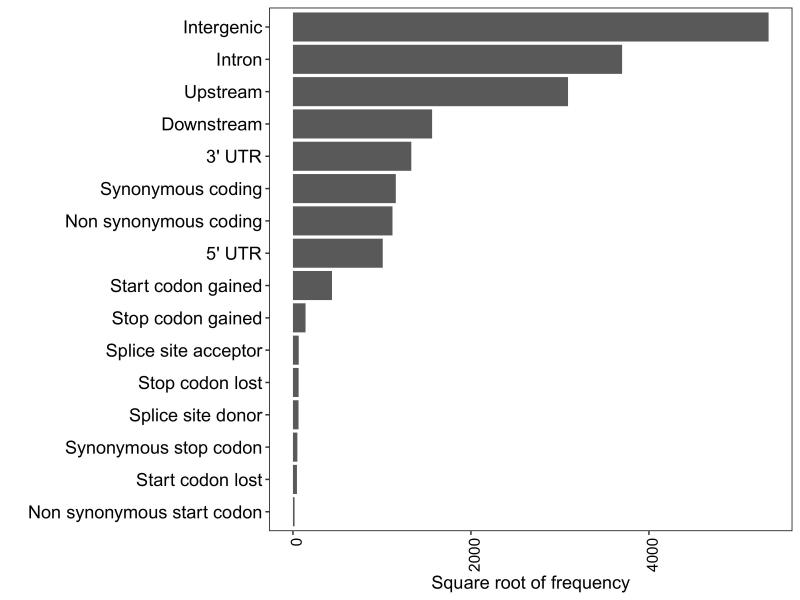
The frequency of SNP sites with varying effects to the genomic structure of the maize reference genome.

The genotype data among 1210 maize lines reported by the maize HapMap 3 project were downloaded from Panzea using iCommands (https://www.panzea.org/genotypes) [2]. The 1210 maize lines were consisted of 1111 improved maize lines, 25 maize landraces, 20 maize wild relatives and 54 other type of maize lines [2]. The raw datasets stored the genotype likelihoods of each maize line across all variation sites, which were converted into genotypes consisting of A, C, G and T using in-house R scripts. InDels were removed and only biallelic SNPs were retained. SNP sites with the number of minor genotypes plus heterozygote genotypes smaller than 5 were filtered out. SNP sites with missing rate higher than 5% were further removed. As a result, 35,370,939 SNP sites were obtained. The distribution of the number of heterozygous genotype and missing genotype across all SNP sites are shown in Fig. S1. Then the maize genome was split into 10-Mb non-overlapping genomic regions and genotype data at SNP sites in each genomic region was stored as an R data file, which could be efficiently loaded into the memory using the R programming language [13]. To facilitate the storage and retrieve of the dataset, the genotype data were converted into integer sparse matrix in the R data files utilizing the approach reported in a recent study [14]. The effect of all SNP sites to the genomic structure of the maize B73 reference genome were annotated using SnpEff [15]. More than 47.7% SNPs were in the intergenic regions while more than 22.8% SNPs were in the intron of genes (Fig. S2).

### 2.2 Construction of the MaizeSNPDB database

R is a prominent language widely used in the analysis of biological data [13]. The Shiny package was designed for R users to make interactive web applications with no knowledge on HTML or JavaScript required, which has been utilize to build complex biological database or web servers successfully [14], [16]. The R Shiny package was utilized in this study to develop the infrastructure and the graphical user interface of MaizeSNPDB. Genotypes of 1210 maize lines across 35,370,939 SNP sites were stored as R data files in MaizeSNPDB, which could be efficiently retrieved without the using of SQL language. The graphical user interface of MaizeSNPDB was enhanced with the help of several R packages including shinythemes, shinyBS, shinycssloaders and shinysky. The power of R could be fully utilized as many genetic algorithms and statistics methods for SNP data analysis were implemented as R packages publicly available. In addition, we also provided several functional modules for investigation of SNPs in user-specified genomic regions in the MaizeSNPDB database, including browsing of SNP sites in user-specified genomic regions, representation of linkage disequilibrium using heatmap, sequence diversity calculation, phylogenetic analysis and bulk download of SNP data, etc.

### 2.3 Interface and functionalities of the MaizeSNPDB database

**Fig. 1:**
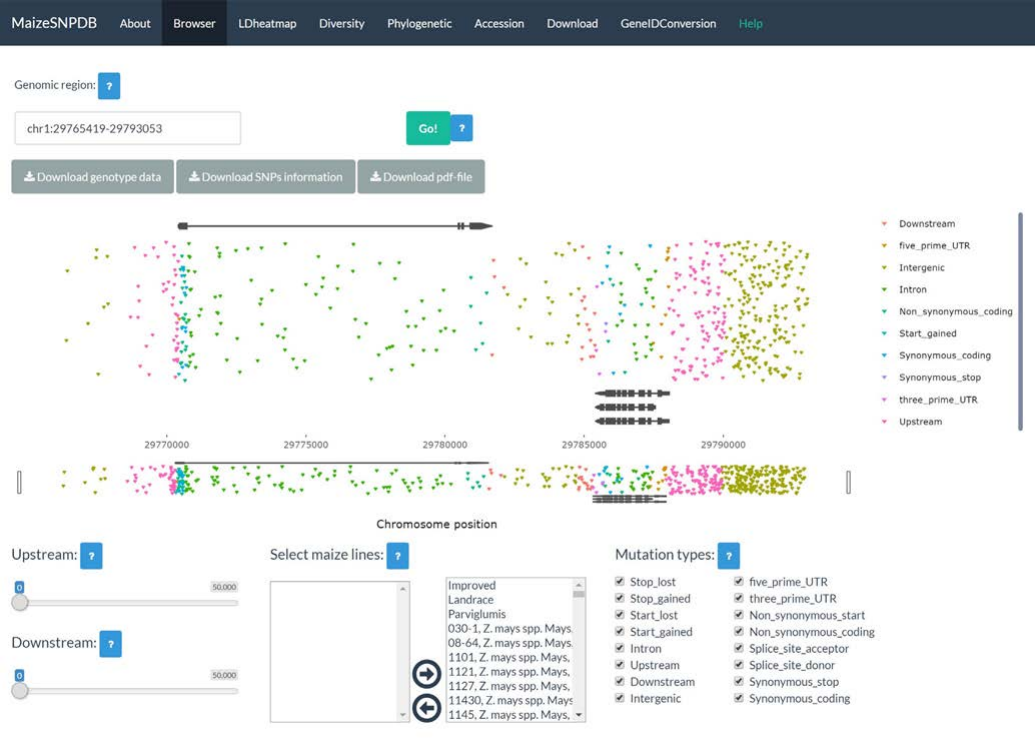
The graphical interface of the MaizeSNPDB database. A total of 8 menus for different functionalities are implemented in the database. The “Browser” menu is shown to illustrate the various widgets, which are designed to receive with user input. A text-input widget is provided for users to specify the genomic region to be visualized. Inverted triangles with differing colors in the main plot region represent SNP sites in user-specified genomic region. Three download widgets on top of the main plot region is designed for downloading of data in the specified genomic region. Three widgets below the main plot region is provided for users to screen the SNP sites displayed in the genome browser. (For interpretation of the references to colour in this figure legend, the reader is referred to the web version of this article.)

The MaizeSNPDB database contains 9 menus, named as “About”, “Browser”, “LDheatmap”, “Diversity”, “Phylogenetic”, “Accession”, “Download”, “GeneIDConversion” and “Help” (Fig. 1). The “About” menu gives a brief introduction of the MaizeSNPDB database and lists all the R packages used in the MaizeSNPDB database. The “Help” menu displays the online use and the installation of the MaizeSNPDB database. The “Accession” menu provides essential information on the 1210 maize lines used in the MaizeSNPDB database. The rest menus of MaizeSNPDB represent the analysis functionalities provided in the database, elaborated in the following section.

**Supplementary Figure 3:**
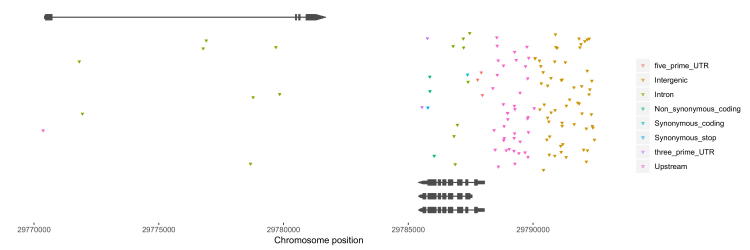
SNPs between two maize lines identified using the genome browser functionality of MaizeSNPDB. SNP sites are represented as inverted triangles with differing colors in the main plot region. Structure of four annotated mRNAs are displayed at the top and bottom of the main plot region. X-aixs, coordinates of the genomic region.

The “Browser” menu provides the functionality to visualize all the SNP sites in a specific genomic region. For a user-specified genomic region or gene model, data of all SNP sites in this region stored as R data files in MaizeSNPDB were loaded into the memory. Then each SNP site was displayed as an inverted triangle in the main plot region of the genome browser (Fig. 1). The position of each triangle in the main plot region was defined by the genomic coordinate of the SNP site (X-axis) and a random assigned value in [0, 1] (Y-axis). The annotated gene models in the genomic region are also displayed on the top or bottom of the genome browser. The genome browser functionality was realized using the ggplot2 and plotly packages in R [17], [18]. The genomic position and effect of each SNP site were shown beside each triangle on mouse-hover. Users can choose to display SNP sites among a subset of maize lines or SNP sites with specific effect to genomic structures using the widgets provided below the main plot region of the genome browser (Fig. 1). In addition, this functionality can be used to visualize and extract all the SNPs between two specified maize lines in a specified genomic region (Fig. S3). The basic information and the genotype of chosen maize lines across all the SNP sites in specified genomic regions could be downloaded as plain text file for further analysis by other tools. The interactive visualization of SNPs in user-specified genomic regions can also be downloaded as static PDF files.

**Fig. 2:**
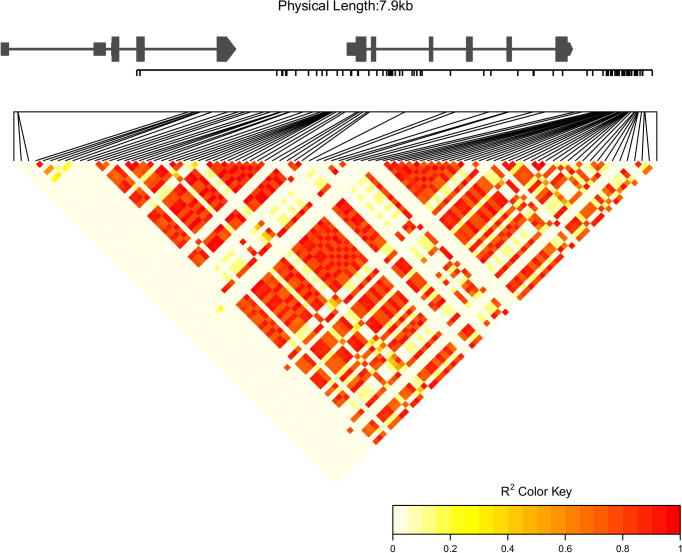
An example linkage disequilibrium heatmap created using the functionality of MaizeSNPDB. Linkage disequilibrium measured in *r*^2^ is represented as an inverted-triangle heatmap. The color of different cells of the heatmap denotes the value of *r*^2^ as indicated in the color key. The structure of annotated gene models is displayed on top of the heatmap. The positions of SNP sites are represented as black ticks along the gene models. (For interpretation of the references to colour in this figure legend, the reader is referred to the web version of this article.)

**Supplementary Figure 4:**
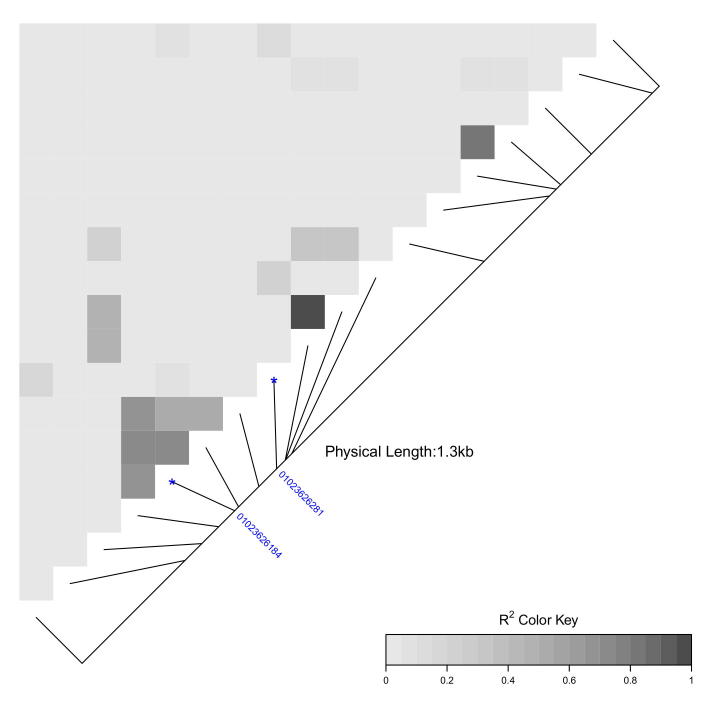
Linkage disequilibrium between SNPs in the genomic region of the gene *gt1* displayed as a heat map.

The “LDheatmap” menu provides the functionality to calculate and display the linkage disequilibrium (LD) between all pairs of SNP sites in a specified genomic region. Linkage disequilibrium heatmap is widely used in the demonstration of local GWAS signals. For a user-specified genomic region or gene model, the linkage disequilibrium measured in *r*^2^ between any pair of SNP sites were calculated and displayed as a heatmap using the R LDheatmap package (Fig. 2) [19]. Maize lines and SNP effect could be used to restrict the SNP sites used in the calculation. Two typical set of colors were provided for the generated heatmap (Fig. 2, Fig. S4). The structure of annotated gene models in the specified genomic region could also be displayed on top of the heatmap (Fig. 2). This functionality can be used to identify block of SNPs in strong LD.

**Fig. 3:**
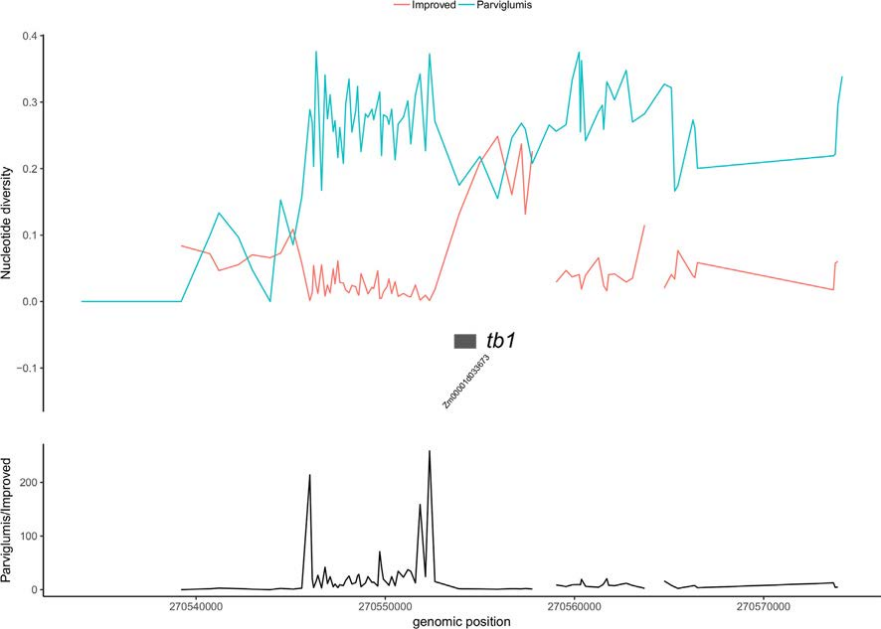
Sequence diversity in wild and modern maize lines around the genomic region harboring the *tb1* gene. The top panel shows the nucleotide diversity among wild maize lines (*Z. mays* ssp. *parviglumis*) and modern maize lines (improved) indicated by different colors. The bottom panel demonstrate the division of nucleotide diversity in the two populations. X-axis, coordinates of the genomic region around *tb1* (Zm00001d033673). (For interpretation of the references to colour in this figure legend, the reader is referred to the web version of this article.)

The “Diversity” menu provides the functionality to calculate nucleotide diversity among group of maize lines in user-specified genomic regions. Shrink of sequence diversity in cultivars compared with that of wild ancestors is an important signal of artificial selection in domestication [3]. A user-specified genomic region was split into non-overlapping genomic regions with each genomic region containing 10 SNP sites (default value). Then the sequence diversity among maize lines belonging to a specific subgroup was calculated for each genomic region using the pegas R package and displayed as connective lines using the ggplot2 package [17], [20]. Gene models in the specified genomic region were also displayed in the plot. The calculated nucleotide diversity could be downloaded as a plain text file for use in other tools. We further provide the functionality to compare the sequence diversity in user-specified genomic regions between different subgroups under the “Diversity” menu of the MaizeSNPDB database. Using this functionality, we show that the promoter region of *tb1* gene was under strong selection during maize domestication, in accordance with the results of previous studies (Fig. 3) [21].

**Fig. 4:**
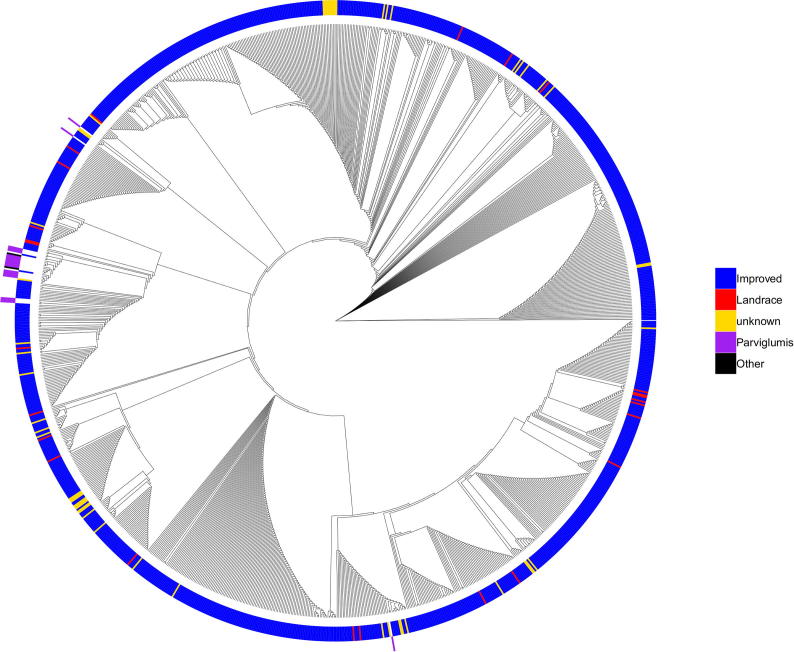
NJ tree constructed based on SNPs around the *tb1* gene using the phylogenetic functionality of MaizeSNPDB. The NJ tree is shown in circular format using the ggtree package. Each tip of the tree represents a maize line. Colors in the inner circle denote the cultivated maize lines while colors in the outer circle indicate the wild maize lines. (For interpretation of the references to colour in this figure legend, the reader is referred to the web version of this article.)

The functionality to perform phylogenetic analysis was implemented in the “Phylogenetic” menu of the MaizeSNPDB database (Fig. 4). Phylogenetic analysis of populations based on genotype data in local genomic regions is helpful to the identification of sequence introgressions, deduction of gene evolution, etc. For a user-specified genomic region or a gene model, the genotype data of all maize lines across all SNP sites in this region were retrieved. The genetic distance between different maize lines was then calculated based on the genotypes across all SNP sites using the allele sharing distance defined in a previous study using in-house R script [22]. Then the APE R package was used to construct the neighbor-joining (NJ) tree based on the genetic distances [23]. The NJ tree was displayed in circular format using the ggtree package [24].

### 2.4 Analysis of artificial selection on maize domestication genes using MaizeSNPDB

Modern maize (*Zea mays* subsp. *mays*) was domesticated from teosinte (*Z. mays* subsp. *parviglumis*) [25]. During this process, several key agronomic traits were altered, leading to the striking phenotypic differences between modern maize and teosinte. Underlying the phenotypic changes is the artificial selection acts on key genes regulating specific agronomic traits. The selection resulted in the expansion of the alleles that are favorable to human beings in modern maize, which further led to the reduction of nucleotide diversity in the genomic regions harboring the genes under selection. The SNP data of 1111 improved maize lines, 25 maize landraces, 20 maize wild relatives were stored in the MaizeSNPDB database. In addition, a graphical interface was developed to calculate and visualize the nucleotide diversity in specified genomic regions. Users can utilize the MaizeSNPDB database to analyze the selection on specific genes very easily.

**Supplementary Figure 5:**
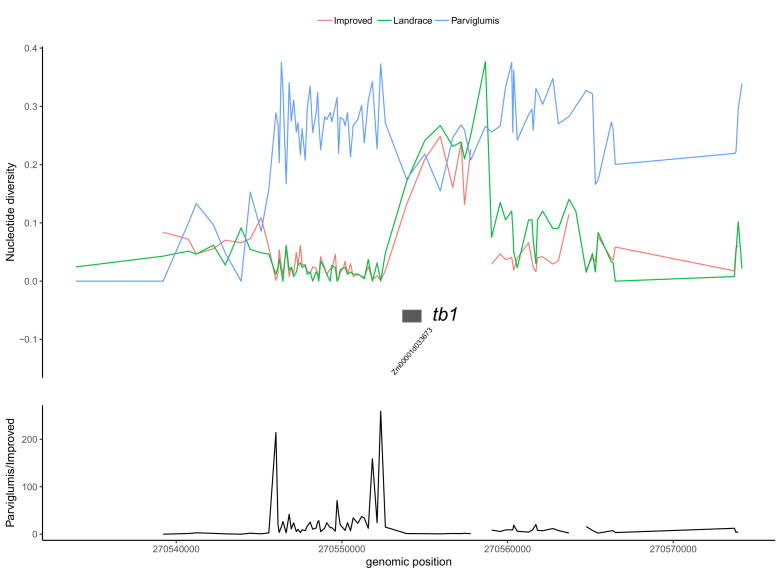
Nucleotide diversity analysis in the genomic region of *tb1*. The top panel shows the nucleotide diversity of different groups of maize lines. The bottom panel shows the division of nucleotide diversity of teosinte lines by that of the improved maize lines. Improved, improved maize lines. Landrace, maize landrace. Parviglumis, *Z. mays* ssp. *parviglumis* (teosinte lines).

**Supplementary Figure 6:**
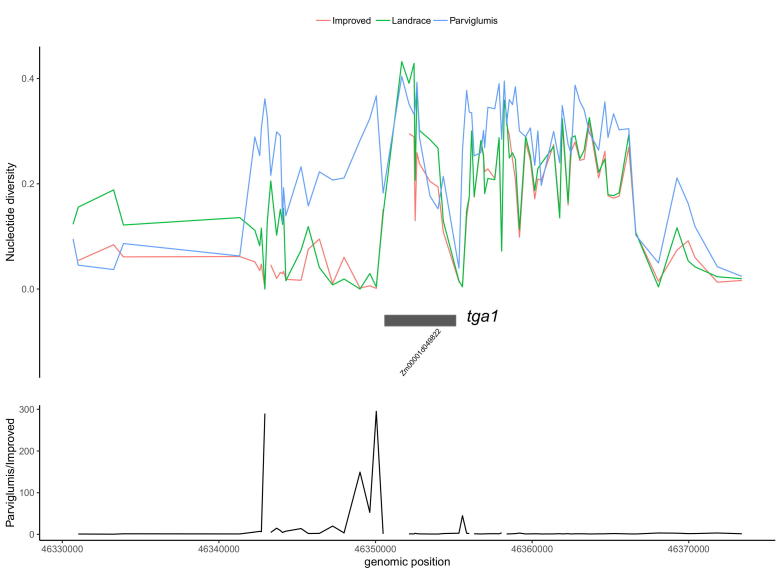
Nucleotide diversity analysis in the genomic region of *tga1*. The top panel shows the nucleotide diversity of different groups of maize lines. The bottom panel shows the division of nucleotide diversity of teosinte lines by that of the improved maize lines. Improved, improved maize lines. Landrace, maize landrace. Parviglumis, *Z. mays* ssp. *parviglumis* (teosinte lines).

**Supplementary Figure 7:**
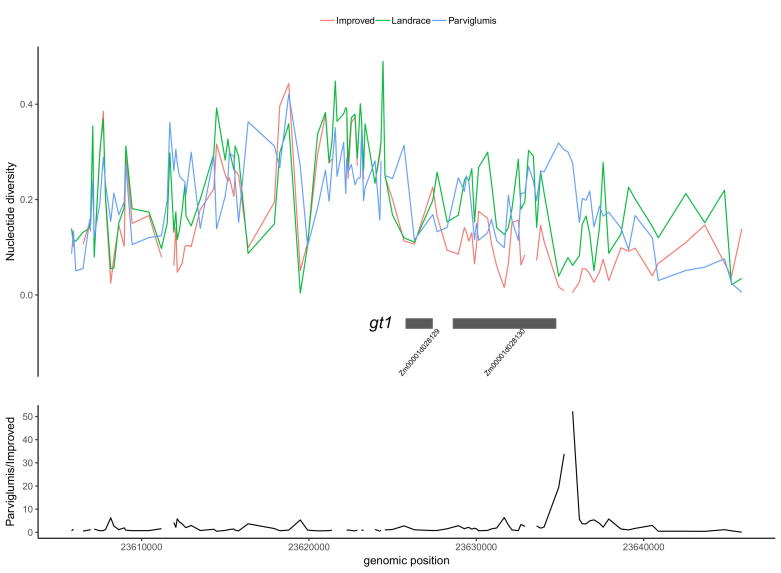
Nucleotide diversity analysis in the genomic region of *gt1*. The top panel shows the nucleotide diversity of different groups of maize lines. The bottom panel shows the division of nucleotide diversity of teosinte lines by that of the improved maize lines. Improved, improved maize lines. Landrace, maize landrace. Parviglumis*, Z. mays* ssp. *parviglumis* (teosinte lines).

*tb1* is a plant architecture gene conferring the change from a highly branched plant to a less-branched plant during maize domestication [21]. The nucleotide diversity in the promoter region of *tb1* was greatly reduced among improved maize lines and maize landraces compared with that of teosinte (Fig. S5). A previous study identified a gene network acting downstream of *tb1* during maize domestication, including *tga1*, *not1*, *zag1*, *gt1* and *ZmMADS2* [25]. The reduction of nucleotide diversity in the promoter region of *tga1* during maize domestication was reported in a previous study and verified by the analysis result of the MaizeSNPDB database (Fig. S6) [26]. However, no significant difference was observed between the nucleotide diversity among improved maize lines v.s. that among teosinte in the genomic region surrounding *gt1*, which controls the number of ears in maize domestication (Fig. S7). This observation agrees with the findings of a previous study, demonstrating the accuracy of SNP data in the MaizeSNPDB database [27].

## 3 Conclusions

**Supplementary Figure 8:**
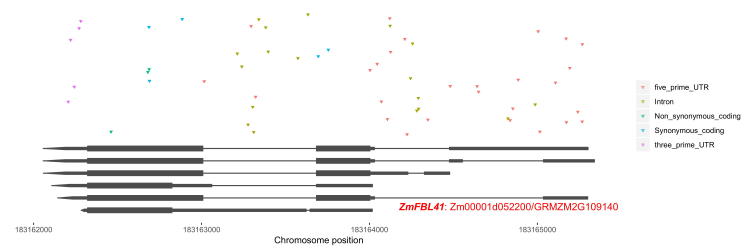
SNPs located within the genic region of *ZmFBL41* demonstrated by the genome browser functionality of MaizeSNPDB. Structure of six annotated mRNAs of *ZmFBL41* are displayed at the bottom of the main plot region. X-aixs, coordinates of the genomic region.

Biological studies in model organisms benefit a lot from the availability of many databases of various usage. We constructed MaizeSNPDB, a SNP database for a large set of maize lines using the genotype data reported by the maize HapMap 3 project [2]. The dataset stored in the MaizeSNPDB database is the most comprehensive genomic variation dataset for maize to date. This database would be helpful to future studies of maize including population genomics studies, marker-assisted breeding and genetic analysis of specific genes, etc. As the functions of more and more maize genes are decoded by map-based gene cloning and GWAS [28], we can use MaizeSNPDB to identify the maize lines with beneficial alleles of specific genes and use these lines as potential donors in molecular breeding. Two or more SNPs flanking the functional variant site can be further utilized to design molecular markers used to validate the introgression of beneficial alleles from the donor maize line to the acceptor maize line in marker-assisted breeding. Recently, a maize disease resistance gene *ZmFBL41* was identified by GWAS [28]. The SNPs reported to be located within the second exon of *ZmFBL41* with the most significant association signals are also present in the MaizeSNPDB database (Fig. S8). Users can investigate the natural variations in *ZmFBL41* with a much larger maize population provided by the MaizeSNPDB database compared with the number of maize lines used in the original study which dissect the function of *ZmFBL41*.

The data provided by the maize HapMap 3 project is the likelihoods of different genotypes in VCF format rather than the deduced genotypes [2]. The whole dataset takes a large amount of disk space. In addition, variation sites with very high missing rate were retained in the result of maize HapMap 3 project. The utilization of the whole dataset is challenging for users without professional coding skills. We refined a subset of high-quality SNP sites from the whole dataset, which were stored as R data files taking small amount of disk usage. We also developed a database for users to browser and extract SNP sites in specified genomic regions, which could be further analyzed using other tools. The database is deployed on Linux web server and can be accessed through the web browser. This would be helpful for in-depth exploitation of this dataset. Users can choose to visualize and download the SNP data between two maize lines, which is helpful to determine the candidate genes underlying specific agronomic trait in map-based gene cloning or genome-wide association mapping.

Although many bioinformatics tools have been developed for the analysis of SNP data, most of these tools are designed for using in command lines or in specific programming languages. In the MaizeSNPDB database, several popular genetic analysis programs were implemented with graphical interface, which can be easily used to perform genetic analysis of specific genes or genomic regions input by the users. Many widgets were implemented in MaizeSNPDB to receive user input. Publication-quality figures could be easily generated and decorated using the tools developed in MaizeSNPDB. The genome annotation of the maize B73 reference genome has been upgraded from version 3 to version 4. A tiny tool for conversion of gene IDs between version 3 and version 4 is implemented in MaizeSNPDB using data from MaizeGDB (https://maizegdb.org/) [29]. These features further enhanced the application of the MaizeSNPDB database, as all the browsing and analysis of data in MaizeSNPDB could be conducted with the graphical interface provided. MaizeSNPDB could be easily deployed on local personal computers or Linux servers.

The MaizeSNPDB database was constructed utilizing the integer sparse matrix approach proposed in a previous study [14]. The database was built using pure R code while the traditional approach to build a database usually involved multiple programming languages. All the code and datasets of the database are deposited in GitHub, which could be applied to construct similar database very easily.

As MaizeSNPDB was created using the R/Shiny framework rather than the common relational database management system, the response of the database could possibly slow down when it was accessed by a very large number of users at the same time. Nevertheless, the R/Shiny framework is adequate for constructing biological applications with not very large amount of user-access at the same time. MaizeSNPDB database was designed to provide rapid genetic analysis of maize genes or genomic regions harboring candidate genes screened from upstream studies. Further experiments are needed to illuminate the genetic and molecular functions of target genes.

## 4 Materials and methods

### 4.1 Genotype data processing

The raw genotype data were downloaded from Panzea using iCommands (https://www.panzea.org/genotypes). InDels or non-biallelic SNPs were removed using the Linux command line tools. Conversion of genotype likelihoods into character genotypes and the filtering of low-quality SNP sites were conducted using in-house R scripts [13]. Finally, a total of 35,370,939 SNP sites were retained. The character genotypes of 1210 maize lines at the 35,370,939 SNP sites were converted into integer sparse matrices composed of 0, 1 and 2, by coding the major homozygous genotype, the minor homozygous genotype and the heterozygous genotype of each SNP site as “0″, ”1″ and “2″ respectively. The R script used to perform the conversion process is provided in the source code of MaizeSNPDB (https://github.com/venyao/MaizeSNPDB/tree/master/script). The sparse matrices implementation in the Matrix package was utilized to store the integer sparse matrices [13].

### 4.2 Constructing the graphical interface and analysis functionalities of MaizeSNPDB

The graphical interface and the analysis functionalities of MaizeSNPDB were realized using several R scripts. The ui.R script defines the graphical interface of MaizeSNPDB and many widgets to accept user-inputs while the server.R script invokes other R scripts to extract and analyze the genotype data in specific genomic regions. The shinythemes package (https://rstudio.github.io/shinythemes/) was used to control the overall appearance of an R/Shiny application and the “flatly” theme provided by the shinythemes package was adopted in the MaizeSNPDB database. Each of the analysis functionalities provided in the MaizeSNPDB database was implemented in a menu of the MaizeSNPDB Shiny application. Each analysis functionality was implemented as an independent R function stored in an R script file. For a user-specified genomic region, an R script was used to extract the character genotype data of 1210 maize lines across all SNP sites in this region. The extracted genotype data would then be subjected to further analysis by different functionalities.

## Availability of supporting source code and requirements

Project name: MaizeSNPDB.

Project home page: http://150.109.59.144:3838/MaizeSNPDB/

GitHub repository: https://github.com/venyao/MaizeSNPDB.

Operating system(s): Platform independent.

Programming language: R (≥3.1.0).

Other requirements: tested with R packages shiny (1.2.0), shinythemes (1.1.2), shinyBS (0.61), IRanges (2.16.0), plyr (1.8.4), plotly (4.8.0), LDheatmap (0.99.5), chopsticks (1.48.0), foreach (1.4.4), APE (5.2), pegas (0.11), ggplot2 (2.2.1), dplyr (0.7.4), tidyr (0.8.0), ggtree (1.10.5), genetics (1.3.8.1) and snpStats (1.28.0).

License: GPLv3.

Any restrictions to use by non-academics: None.

## Declaration of Competing Interest

The authors declare that they have no known competing financial interests or personal relationships that could have appeared to influence the work reported in this paper.
